# Breaking bread: examining the impact of policy changes in access to state-funded provisions of gluten-free foods in England

**DOI:** 10.1186/s12916-018-1106-7

**Published:** 2018-08-02

**Authors:** Myles-Jay Linton, Tim Jones, Amanda Owen-Smith, Rupert A. Payne, Joanna Coast, Joel Glynn, William Hollingworth

**Affiliations:** 1 Health Economics at Bristol, Population Health Sciences, Bristol Medical School, University of Bristol, Bristol, UK; 2 The National Institute for Health Research Collaboration for Leadership in Applied Health Research and Care West (NIHR CLAHRC West), University Hospitals Bristol NHS Foundation Trust, Bristol, UK; 3 Centre for Academic Primary Care, Population Health Sciences, Bristol Medical School, University of Bristol, Bristol, UK

**Keywords:** Gluten-free, Coeliac disease, Prescriptions, Health expenditures, Primary health care, National Health Service, Deprivation, Clinical commission groups

## Abstract

**Background:**

Coeliac disease affects approximately 1% of the population and is increasingly diagnosed in the United Kingdom. A nationwide consultation in England has recommend that state-funded provisions for gluten-free (GF) food should be restricted to bread and mixes but not banned, yet financial strain has prompted regions of England to begin partially or fully ceasing access to these provisions. The impact of these policy changes on different stakeholders remains unclear.

**Methods:**

Prescription data were collected for general practice services across England (*n* = 7176) to explore changes in National Health Service (NHS) expenditure on GF foods over time (2012–2017). The effects of sex, age, deprivation and rurality on GF product expenditure were estimated using a multi-level gamma regression model. Spending rate within NHS regions that had introduced a ‘complete ban’ or a ‘complete ban with age-related exceptions’ was compared to spending in the same time periods amongst NHS regions which continued to fund prescriptions for GF products.

**Results:**

Annual expenditure on GF products in 2012 (before bans were introduced in any area) was £25.1 million. Higher levels of GF product expenditure were found in general practices in areas with lower levels of deprivation, higher levels of rurality and higher proportions of patients aged under 18 and over 75. Expenditure on GF food within localities that introduced a ‘complete ban’ or a ‘complete ban with age-related exceptions’ were reduced by approximately 80% within the 3 months following policy changes. If all regions had introduced a ‘complete ban’ policy in 2014, the NHS in England would have made an annual cost-saving of £21.1 million (equivalent to 0.24% of the total primary care medicines expenditure), assuming no negative sequelae.

**Conclusions:**

The introduction of more restrictive GF prescribing policies has been associated with ‘quick wins’ for NHS regions under extreme financial pressure. However, these initial savings will be largely negated if GF product policies revert to recently published national recommendations. Better evidence of the long-term impact of restricting GF prescribing on patient health, expenses and use of NHS services is needed to inform policy.

**Electronic supplementary material:**

The online version of this article (10.1186/s12916-018-1106-7) contains supplementary material, which is available to authorized users.

## Background

Healthcare services across Europe are under increasing financial pressure [1]. In 2015/2016 England’s state-funded National Health Service (NHS) deficit reached £1.85 billion, the largest deficit in NHS history [2]. Despite a subsequent reduction in the deficit in 2016/2017 [3], there is ongoing pressure to achieve the NHS Five Year Forward View’s target of finding savings of £22 billion by 2020 [4]. These financial difficulties prompt healthcare decision-makers to reconsider what the NHS can afford to provide. A widely noted reaction to this pressure has been the increasing prioritisation of cost-saving in the management of medicines, and subsequent disinvestment in prescribed items deemed to be of ‘low clinical value’ such as travel vaccines and homeopathy [5]. Another highly debated example is the prescription of gluten-free (GF) foods for people with gluten enteropathy, also known as coeliac disease (CD) [6].

CD is an autoimmune disease characterised by gastrointestinal symptoms following the ingestion of grains containing gluten (wheat, barley and rye) [7, 8], which affects approximately 1% of the population of Europe [9]. There was a four-fold increase in the incidence rate of CD recorded in the UK between 1990 and 2011, which was largely attributed to increased availability of routine diagnostic tests and greater awareness among clinicians and patients [10]. Like many autoimmune diseases [11], CD is diagnosed at a higher rate among women [10]. There is also evidence that the diagnosis of CD is higher within populations with lower levels of socioeconomic deprivation [12].

Unlike other autoimmune diseases, the only clinically effective treatment for CD is lifelong adherence to a GF diet [13], which is likely to be more financially burdensome than purchasing conventional products that contain gluten [14, 15]. Healthcare systems across the globe have approached this challenge in a variety of different ways. For example, people with CD in Canada are eligible for tax reductions to compensate for the additional costs of purchasing GF foods, while in Italy they are provided with a monthly cash allowance [16]. In the UK, the NHS has provided GF foods on prescription since the 1960s [17]. NHS prescriptions are available for a range of staple goods such as bread and pasta, along with sweet products such as cakes and biscuits [18]. A study into CD prevalence in the UK found that 80% of patients diagnosed with CD were in receipt of at least one GF prescription product through the NHS [10].

In February 2018, NHS England released the results of a nation-wide consultation and announced that prescriptions for GF foods should be restricted to bread and mixes (which can be used to make bread products such as rolls and loaves) [19]. The policy review was prompted by the increased accessibility of GF foods, in comparison to when GF food prescriptions were first introduced [20]. However, prior to this policy decision, an increasing number of NHS England’s Clinical Commissioning Groups (CCGs) had already begun to fully or partially cease funding for prescriptions of GF products in some localities [21]. In sum, the prescription of clinically effective and widely available food products highlights a grey area concerning what should (and what should not) be considered ‘medicine’.

The overarching aim of the study was to investigate the impact of potential policy changes on different stakeholders (patient groups and CCGs), by exploring three distinct objectives. First, we described changes in expenditure on GF products across all CCGs in England between 2012 and 2017. Next, we estimated GF food prescribing expenditure (for 2014) by general practitioner (GP) practice demographics, rurality and deprivation to identify groups of patients likely to be affected most by reduced prescribing of GF products. Finally, in a separate analysis, we compared the cost-savings made by CCGs which have switched to a ‘complete ban’ or a ‘complete ban with age-related exceptions’ with CCGs that have continued to provide GF product prescriptions.

## Methods

### Datasets

#### Prescribing data

Prescriptions for GF products were accessed through ‘ OpenPrescribing.net, EBM DataLab, University of Oxford, 2017’, a resource that organises and presents the raw anonymised prescription data released by NHS Digital each month. These data are based on all NHS prescriptions dispensed in primary care pharmacies in England. For this study, OpenPrescribing provided us with a custom extract of the data containing the ‘actual cost’ of spending on GF products for all GP practices within England (grouped by CCG). NHS Digital defines ‘actual cost’ as the cost to the NHS, rather than the basic price of a drug or product (Net Ingredient Cost). The extract contained data on spending by each GP practice for each month from January 2012 through to June 2017. GF products were classified into three categories as bread products, staple products (e.g. flour and pasta) and other products (e.g. snacks and biscuits). A complete list of the products in each category is presented seperately (Additional file 1).

#### GP practice characteristics

We used data from NHS Digital from 2014 to calculate the percentage of registered patients who were female for each GP practice [22]. We used Public Health England’s National General Practice Profiles (2014) to characterise GP practices in terms of, percentage of registered patients under 18 years of age, percentage of registered patients 75 years of age or older, population size (list size), Indices of Multiple Deprivation (IMD) score from 2015, and parent CCG [23]. By linking each GP practice postcode to a Lower Layer Super Output Area we were able to classify each GP practice by its level of rurality [24]. Specific Lower Layer Super Output Area rurality classifications were collated into broader categories as follows: Urban Conurbation (A1 and B1), Cities and Towns (C1 and C2), and Rural (D1, D2, E1 and E2). Finally, a spending rate was calculated for each GP practice as the total spend on GF prescriptions in 2014 per 1000 registered patients. We selected this time period as it was the year before most NHS CCGs began restricting their GF food policies. Of the 7596 practices in the 2014 GP prescribing data, 7176 (94%) had complete data once all datasets were linked on GP practice code. One further practice was excluded because more than 80% of its patients were aged above 75.

#### CCG policies

Data on CCG policies for GF prescribing were extracted from the Coeliac UK website [21]. After contacting all 207 CCGs, Coeliac UK reviewed policies for GF provisions as part of their ‘Prescriptions Campaign’. This work resulted in an interactive web-based map containing CCG policies across England. Coeliac UK groups CCGs into four policy types, as (1) partial or complete withdrawal of prescriptions; (2) following national prescribing guidelines; (3) restricting products and/or units; and (4) policy or GF prescribing under review. Within this study these policy types were re-categorised into (1) complete ban on prescriptions, (2) complete ban on prescriptions (with age-related exceptions), (3) no ban on prescriptions, (4) partial restrictions on products and/or units, and (5) policy under review. The rationale for re-classifying the policies, particularly ‘(1) partial or complete withdrawal of prescriptions’ was to recognise the meaningful difference between a restriction and a complete withdrawal of provisions. Verification of Coeliac UK’s policy data against the information available on CCG websites was conducted for a small, random selection of CCGs (*n* = 21, 10%). For each of these CCGs, Coeliac UK’s data accurately reflected the policy arrangements stated in official statements on CCG websites.

The mapping between these policies is presented separately (Additional file 2). All policy data were extracted by the research team (on 05/09/2017) and stored in a spreadsheet. A summary table of the frequency of the five policy types is presented in Table 1. The highest number of CCGs followed a ‘partially restricted’ policy, limiting the number of units and products available for prescription (70/207, 34%).

**Table 1:** Summary of Clinical Commissioning Group gluten-free prescribing policies in 2017

Policy type	Frequency (%)
Partial restrictions on products and/or units^a^	70 (34%)
No ban	61 (29%)
Complete ban	46 (22%)
Policy under review	18 (9%)
Complete ban (with age-related exceptions)^b^	12 (6%)

^a^Prescribing was limited to a lower number of monthly units or a restricted set of products which was largely dependent on the individual Clinical Commissioning Group

^b^Gluten-free products still prescribed for children/adolescents under the age of 18 or 19, and in some cases pregnant women

Over one-quarter of CCGs had introduced a ‘complete ban’ or ‘complete ban with age-related exceptions’ (58/207, 28%). Due to the variability in policy details among CCGs with partial restrictions, and availability of data on CCGs with policies under review, this study focused on CCGs with no ban, a complete ban, or a complete ban (with age-related exceptions). Five bans were introduced in 2015 (complete bans = 4, complete ban with age-related exceptions = 1), 22 in 2016 (complete bans = 17, complete ban with age-related exceptions = 5), and 28 were introduced in the first two-quarters of 2017 (complete bans = 16, Complete ban with age-related exceptions = 12).

### Statistical analysis

Total spending for each quarter was calculated across all CCGs (*N* = 207) in England between 2012 (Quarter 1 – January, February and March) and 2017 (Quarter 2 – April, May and June). Total spending was split into the three main product categories (bread products, staple products and other products).

To estimate GF prescribing expenditure by GP practice demographics, rurality and deprivation, GP practices with complete data (for 2014) were entered into a multi-level gamma regression, using the ‘meglm’ command in Stata (version 14.2) with a gamma distribution and log link function. We estimated how spending rate varied with the following predictors: percentage of women, percentage of population aged under 18, percentage of population aged 75+, IMD 2015 (classified into quintiles, where 1 = least deprived), and rural/urban classification. GP practices were clustered by their parent CCG in the model.

To be eligible for inclusion in the examination of policy impacts on spending rates we required CCGs to have GF spending data for at least 3 months following policy changes. Given that the latest available data were for June 2017, CCGs were eligible if they had switched to a complete ban (*n* = 24) or partial ban with age-related exceptions (*n* = 8) prior to April 2017. The process undertaken to match these CCGs to CCGs with a ‘no ban’ policy types was based on annual expenditure in 2014 and is outlined in separately (Additional file 3).

## Results

### Spending on GF products in the past 5 years

**Fig. 1:**
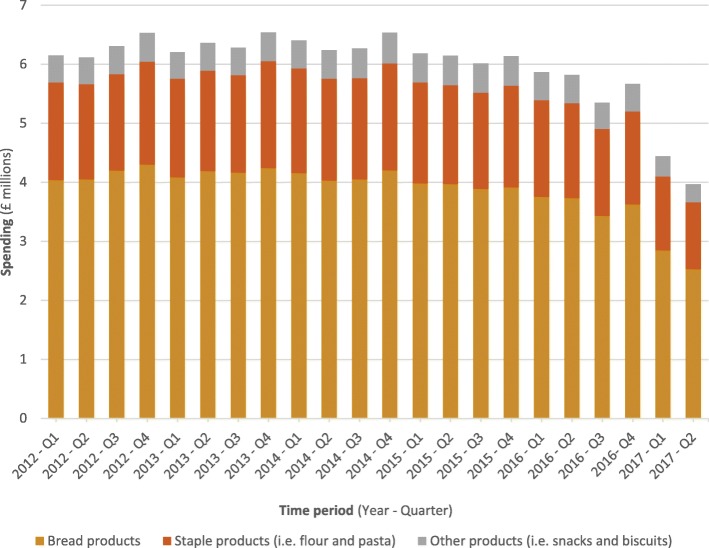
Spending on gluten-free items (2012–2017) across all Clinical Commissioning Groups in England

Annual expenditure on GF products in 2012 (pre-policy bans) was £25.1 million (Fig. 1). Throughout the study period, ‘Bread products’ accounted for the largest proportion (64–67%) of spending. Spending began to decline in 2015 when some CCGs introduced policies to restrict prescribing. The lowest quarterly spending rate (£3.96 million) was observed for 2017 (Quarter 2). This is equivalent to a 39% reduction (quarterly saving of £2.58 million) in overall expenditure since spending on GF products peaked in the fourth quarter of 2014 (£6.54 million).

### The impact of sex, age, rurality and deprivation on GF spending

Regression results for GP practices with complete data in 2014 (*n* = 7175) demonstrated deprivation had a strong association with expenditure (Table 2). GP practices in the most deprived areas (IMD 5th quintile) had the lowest spending rates on GF products (adjusted point estimate 0.77, 95% confidence interval (CI) 0.72–0.83). Incremental reductions in deprivation (lower quintiles) were associated with incrementally higher spending rates on GF products. GP practices in urban conurbations had the lowest spending rates among the three levels of rurality (adjusted point estimate 0.85, 95% CI 0.78–0.93). GP practices with a higher percentage of patients under 18 (adjusted point estimate 1.02, 95% CI 1.02–1.03) and over 75 (adjusted point estimate 1.07, 95% CI 1.06–1.07) had higher spending on GF prescriptions. Sex was not associated with spending on GF prescriptions after adjusting for the remaining variables in the model (adjusted point estimate 1.00, 95% CI 0.99–1.01). As a sensitivity analysis, we examined whether there was an interaction between rurality and deprivation, yet the interaction term was not found to be statistically significant (Additional file 4).

**Table 2:** Modelling spending rate (in 2014), clustered by Clinical Commissioning Group, sex/age variables

Model predictors	Unadjusted point estimate (95% confidence interval)	Adjusted^a^ point estimate (95% confidence interval)
Sex		
Change per % increase in female patients	1.03 (1.02–1.04)	1.00 (0.99–1.01)
Age		
Change per % increase in patients ≤ 18 years	0.99 (0.99–1.00)	1.02 (1.02–1.03)
Change per % increase in patients ≥ 75 years	1.06 (1.05–1.07)	1.07 (1.06–1.07)
Level of rurality		
Baseline: Rural	1	1
Towns and Cities	0.85 (0.81–0.90)	0.93 (0.89–0.98)
Urban Conurbation	0.73 (0.67–0.80)	0.85 (0.78–0.93)
Level of deprivation		
Baseline: 1 – Least Deprived	1	1
2	0.88 (0.83–0.92)	0.93 (0.88–0.98)
3	0.80 (0.76–0.85)	0.88 (0.83–0.93)
4	0.71 (0.67–0.75)	0.80 (0.75–0.85)
5 – Most Deprived	0.67 (0.63–0.71)	0.77 (0.72–0.83)

^a^Adjusted for all other predictors in the model

### Trends in expenditure on GF products within CCGs with alternative GF prescription policies

**Fig. 2:**
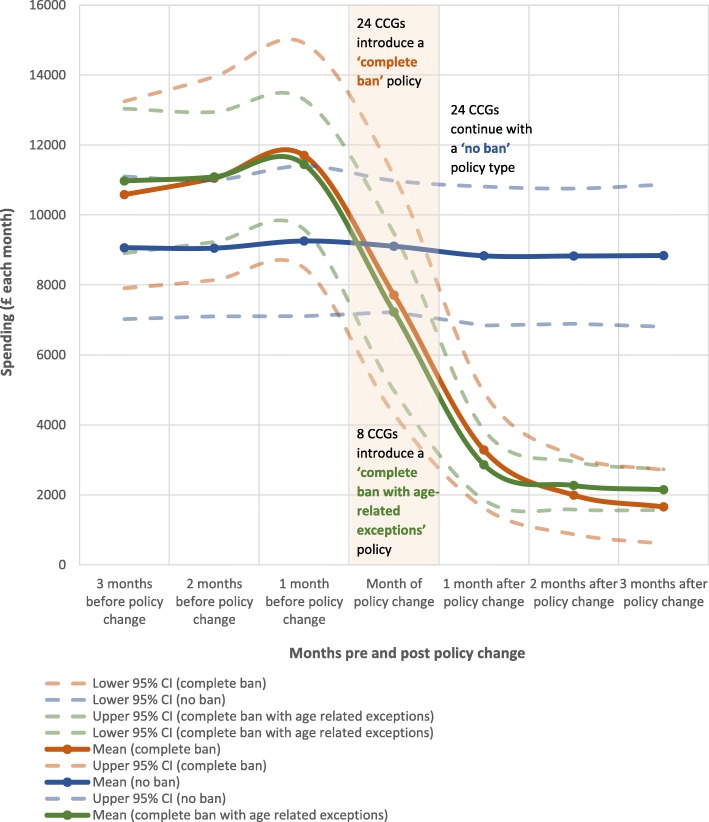
Average (mean) monthly spending within Clinical Commissioning Groups pre (3 months), during, and post (3 months) policy changes

The average total monthly spending rates within CCGs with a ‘complete ban’ policy, were compared to matched CCGs that had ‘no ban’ on GF prescriptions, and to CCGs with ‘complete ban with age-related exceptions’ (Fig. 2). Further details on the spread of these data are presented in Additional file 5. Over time, spending within CCGs with no ban on GF prescribing remained unchanged. In contrast, CCGs that introduced a complete ban saw an average reduction in spending of approximately 80% (equivalent to £9100–£9400 savings per CCG per month). The effect of the policy change had an immediate impact on spending and the reduction in spending levelled off around the second month following policy change. A comparable drop in spending was observed between CCGs which introduced a complete ban and those which had introduced a complete ban for adults only. Despite matching CCGs on their annual expenditure in 2014, expenditure was slightly lower in the ‘no ban’ CCGs in the 3 months leading up to the ban, which might reflect the fact that policy reforms in some areas took some years to implement. If all CCGs had introduced a complete ban on GF prescriptions in 2014, and spending rates followed the 82.9% reduction observed in this study, the NHS in England would have saved £21.1 million on GF products that year. This figure is equivalent to 0.24% of the total expenditure on medicines in primary care for 2014/2015 (£8.7 billion) [25].

## Discussion

### Main findings

We found that expenditure on GF products was reduced by an average of approximately 80% within the 3 months after CCGs introduced a ‘complete ban’ or ‘complete ban with age-related exceptions’ on GF prescriptions. Spending on GF products peaked in the fourth quarter of 2014 (2014 Q4: £6.54 million) and has since fallen by approximately one-third (2017 Q2: £3.96 million). We estimate that if all CCGs had introduced a ‘complete ban’ policy for 2014 the NHS would have made an annual cost-saving of £21.1 million. The policies are likely to have the largest impact on spending among GP practices in the least deprived areas, most rural locations and with the highest proportion of patients over the age of 75.

### Strengths and weaknesses of the study

To our knowledge, this is the first study to examine the impact of GF product prescription policy changes on cost-savings. As such, our work provides timely insight into the financial implications of recent NHS England GF prescription policy options. This study is comprehensive and representative due to its use of prescription data from all 207 CCGs in England. We explored the cost-savings associated with introducing a ‘complete ban’ or ‘complete ban with age-related exceptions’ compared to CCGs with a ‘no ban’ policy. Differences in the specific criteria used by CCGs with a ‘partial restriction on products and/or units’ meant that they were not examined in this analysis. Further, we did not have access to data on the number of people with diagnosed CD in each GP practice. Given our focus on prescription data, this work presents one part of a larger story. For example, it is unclear what the impact of policy changes might be on the frequency of consultations, medications or referral rates for gastrointestinal symptoms.

### Comparison with other studies

In line with evidence that CD is diagnosed at a higher rate among more affluent patients [12], this study found that spending on GF prescriptions was highest in GP practices with the lowest levels of deprivation. One explanation for this may be that symptom recognition and care seeking is higher among people in higher socioeconomic positions [26]. We also found that GF product expenditure was higher within GP practices with higher proportions of patients over the age of 75 and under the age of 18. In England, patients in both of these age categories are exempt from prescription charges, and therefore this observation is expected [27]. Finally, although CD is diagnosed in more women than men [10], having a higher proportion of female patients within GP practices was not associated with greater expenditure on GF products (when controlling for rurality, age and deprivation).

### Implications for patients, clinicians and policymakers

The results of this study demonstrate that policy-driven restrictions on GF prescriptions have been a quick and effective strategy for CCGs to reduce their expenditure. Given our findings, new national recommendations that endorse the prescription of GF bread and mix products are unlikely to yield meaningful cost savings for CCGs [19]. Additionally, the publication of national recommendations 3 years after CCGs began acting unilaterally is likely to create further confusion amongst patients. These changes may be particularly confusing within localities that previously ceased funding for GF products which may now decide to reintroduce them to be in accordance with national recommendations. The higher price paid by the NHS for similar products that would cost less for patients to buy in retail outlets remains a contentious topic [6]: however, higher savings might be yielded if improvements were made to the procurement processes currently in place.

While GF products may be increasingly available in retail stores, they are not necessarily nutritionally equivalent to the products prescribed currently through the NHS. Prescribed products through specialist suppliers such as Juvela [28] and Glutafin [29] are fortified with calcium, iron, folic acid and B vitamins, nutrients that CD patients are commonly deficient in due to malabsorption in the intestines [30]. In the UK, retail wheat flour is typically fortified, yet this is not necessarily the case with GF substitute products [31]. If policy changes result in CD patients switching to retail suppliers for their GF products, they may need to complement their diet with additional nutritional supplements, which may impose additional costs.

Despite technological advancements in the diagnosis of CD, patients often wait many years (mean 5.8 years) following their first GP consultation to receive a formal diagnosis of CD [32], during which there is evidence of increased costs to the NHS [33]. If prescription policies tighten, patients who have faced delayed diagnoses will face the additional obstacle of no access to NHS prescribed treatment.

Restricting GF prescribing may in fact be a false economy. If patients are unable to achieve a GF diet when they do not have GF prescriptions [34], and this leads to an increase in symptoms, then the policies will have a negative effect on NHS resources and health outcomes in the medium term. In addition, the restriction of GF prescribing will have financial consequences for people with CD. Although this study found that spending on GF prescriptions has been highest amongst GP practices in the least deprived areas, those less affluent patients who do access GF prescriptions may be the most likely to struggle with the added financial strain of adhering to a GF diet in the absence of prescriptions [14]. To address this knowledge gap, a qualitative examination into the impact of these policy changes on patients would provide useful insight into how the withdrawal of prescriptions might influence compliance with a GF diet.

In the face of this mismatch between CCG policies and national recommendations, a grey area concerning the extent to which ‘food’ products are considered ‘healthcare’ is revealed. While the link between food and health is widely noted [35–37], including food items such as GF bread alongside pharmaceutical products on the national formulary is, and will continue to be, controversial. This is particularly so given that prescription rates are highest among GPs in more affluent areas, suggesting that GF product prescriptions may not be reducing health inequalities. In the longer term, the solution in England might be for greater cooperation between the government, food manufacturers and the retail industry to make GF products more affordable and widely available.

### Future research

Despite many CCGs already opting to limit the prescription of GF products [21], our understanding of the wider impact of these policy changes remains incomplete. This study explored the short-term financial consequence of policy restrictions, but there is a need to also conduct medium- and longer-term analyses to explore the resource implications of these policy changes in terms of GP consultations, hospital admissions and other forms of healthcare utilisation. Similarly, the impact of policy changes on health outcomes could be highlighted by investigating increases in the use of vitamin and mineral supplements, and gastrointestinal drugs such as anti-diarrhoeals to combat gluten-induced symptoms. Several studies have indicated that GF foods can cost up to four-times as much as gluten-containing alternatives [14, 15]; therefore, there is a need to investigate the financial burden that more restrictive GF food policies shift onto patients.

Interestingly, NHS regions (CCGs) that implemented a complete ban policy did not see a full reduction in expenditure on GF products. Within the 3-month period studied, spending within these CCGs decreased by approximately 80%, yet this also reveals that GF products were still being prescribed and supplied while they were banned. All of the CCGs (apart from one CCG at 2 months post-policy change) reported that expenditure on GF products had not been completely reduced. One explanation for this finding is that there may have been slow or, in some cases, incomplete implementation of policies within CCGs. Further exploration is needed into the personal perspectives of GPs [38], and the exceptional circumstances that might be used as rationale for prescribing banned products. As in other clinical settings, there is a need to further explore the interplay between policy-level decision-making and prescribing decisions made at the patient level [39].

## Conclusions

The introduction of more restrictive GF prescribing policies has been associated with ‘quick wins’ for CCGs under extreme financial pressure. However, these initial savings will be largely negated if CCGs revert to recently published national recommendations. Better evidence of the long-term impact of restricting GF prescribing on patient health, expenses and use of NHS services is needed to inform policy.

## Additional files

Additional file 1: List of gluten-free products in each product category. (DOCX 12 kb)

Additional file 2: Mapping between policy types. (DOCX 20 kb)

Additional file 3: Matching process used for examination into policy-related cost-savings. (DOCX 14 kb)

Additional file 4: Modelling Spending Rate (in 2014), clustered by CCG, sex/age variables. (DOCX 14 kb)

Additional file 5: Mean and standard deviations for expenditure on GF products among CCGs with three policy types. (DOCX 13 kb)

## Abbreviations

CCG: Clinical Commissioning Group
CD: coeliac disease
CI: confidence interval
GF: gluten free
GP: general practitioner
IMD: indices of multiple deprivation
NHS: National Health Service
