# Supplementary material for: Breaking bread: examining the impact of policy changes in access to state-funded provisions of gluten-free foods in England
Source: BMC Med. 2018 Aug 2;16:119. doi: 10.1186/s12916-018-1106-7 (PMC6090920; doi:10.1186/s12916-018-1106-7)
Supplement: Supplementary file 1 — List of gluten-free products in each product category. (DOCX 12 kb) [file 12916_2018_1106_MOESM1_ESM.docx]

## Additional file 1

File name: Additional file 1

File format: Docx

Title of the data: List of gluten-free products in each product category

| **Product type** | **Products** |
| --- | --- |
| Bread products | ‘Gluten-free bread’, ‘gluten-free and low protein bread’ and ‘gluten-free and wheat-free bread’. |
| Staple products | ‘Gluten-free grains/flour’, ‘gluten-free and low protein grains/flour’, and ‘gluten-free and wheat-free grains/flour’.  ‘Gluten-free pasta’, ‘gluten-free and low protein pasta’, ‘gluten-free and wheat free pasta’, and ‘gluten-free, wheat free and low protein pasta’.  ‘Gluten-free mixes’, ‘gluten-free and low protein mixes’, ‘gluten-free and wheat free mixes’, and ‘gluten-free, wheat free and low protein mixes’. |
| Other products | ‘Gluten-free and wheat-free snacks’.  ‘Gluten-free cakes/pastries’ and ‘gluten-free and wheat free cakes/pastries’.  ‘Gluten-free and low protein cereal’ and ‘gluten-free and wheat free cereal’.  ‘Gluten-free and low protein meals’ and ‘gluten-free and wheat free meals’.  ‘Gluten-free and low protein cooking aids’, ‘gluten-free and wheat free cooking aids’, and ‘gluten-free, wheat free and low protein cooking aids’.  ‘Gluten-free biscuits’, ‘gluten-free and low protein biscuits’, ‘gluten-free and wheat free biscuits’, and ‘gluten-free, wheat free and low protein biscuits’. |
