# Supplementary material for: Breaking bread: examining the impact of policy changes in access to state-funded provisions of gluten-free foods in England
Source: BMC Med. 2018 Aug 2;16:119. doi: 10.1186/s12916-018-1106-7 (PMC6090920; doi:10.1186/s12916-018-1106-7)
Supplement: Supplementary file 2 — Mapping between policy types. (DOCX 20 kb) [file 12916_2018_1106_MOESM2_ESM.docx]

## Additional file 2

File name: Additional file 2

File format: Docx

Title of the data: Mapping between policy types

| **Policy labels from Coeliac UK** | **Policy labels in this study** |
| --- | --- |
| (1) Partial or complete withdrawal of prescriptions | (1) Complete ban on prescriptions |
|  | (2) Complete ban on prescriptions (with age related exceptions) |
| (2) Following national prescribing guidelines | (3) No ban on prescriptions |
| (3) Restricting products and/or units | (4) Partial restrictions on products and or units^a^ |
| (4) Policy or GF prescribing under review | (5) Policy under review^b^ |
| **Notes:**  a = at least 12 alternative ‘partial policies’ were identified:   - 1. Bread and plain flour (4 units)   2. Fresh and long-life bread, bread mix and flour (8 units)   3. Restricted list of fresh and long-life bread, rolls, flour and breadmix (National Prescribing Guidelines for quantities)   4. Restricted list of fresh and long-life bread, rolls, flour and breadmix (National Prescribing Guidelines for quantities every 2 months)   5. Fresh and long-life bread, rolls bread mix, flour and pasta from a restricted list (Following national prescribing guidelines for quantities)   6. Breads (fresh and long-life), rolls, flour, bread mix and past (Units reduced by 25% of the national prescribing guidelines)   7. Bread, flour and flour mix from a restricted list (6 -11 units depending on age)   8. Bread, flour, flour mixes and pasta (7 - 13 units depending on age and gender)   9. Bread, flour and bread mixes (9 units)   10. Bread, bread mix, flour, flour mix, crackers and pasta (6-12 units a month dependent on age and gender)   11. 3 units less than the national prescribing guidelines   12. Not prescribing fresh bread, pizza bases, crispbreads, breakfast cereals and oats (Follow national prescribing guidelines for quantities)   b = For CCGs undergoing reviews of their policies, the dataset did not include details on current practices. | |
|  | |
