# Supplementary material for: Breaking bread: examining the impact of policy changes in access to state-funded provisions of gluten-free foods in England
Source: BMC Med. 2018 Aug 2;16:119. doi: 10.1186/s12916-018-1106-7 (PMC6090920; doi:10.1186/s12916-018-1106-7)
Supplement: Supplementary file 3 — Matching process used for examination into policy-related cost-savings. (DOCX 14 kb) [file 12916_2018_1106_MOESM3_ESM.docx]

## Additional file 3

File name: Additional file 3

File format: Docx

Title of the data: Matching process used for examination into policy related cost-savings

We matched 24 CCGs that had introduced a complete ban on GF prescriptions to 24 CCGs which continued to provide GF prescriptions throughout the study period. Matching was undertaken on the basis of annual expenditure in 2014. CCGs were categorised into four quantiles, (ranging from, 1 = CCGs with the lowest GF spending-rates in 2014, to 4 = CCGs with the highest GF spending-rates in 2014). 2014 expenditure was chosen as it was a period before CCGs began introducing GF policies. Each CCG that had introduced a complete ban was matched to one randomly selected CCG from the same 2014 expenditure quartile that had no ban throughout the study period. Each matched pair was assigned an ‘index month’, defined as the month of the policy change in the CCG that introduced the complete ban. Monthly spending rates were calculated for the three months prior to the index month and the three subsequent months. Monthly expenditure was averaged (mean) for CCGs from the three main policy types: (1) Complete ban, (2) Complete ban with age-related exceptions, and (3) No ban.
