# Supplementary material for: Breaking bread: examining the impact of policy changes in access to state-funded provisions of gluten-free foods in England
Source: BMC Med. 2018 Aug 2;16:119. doi: 10.1186/s12916-018-1106-7 (PMC6090920; doi:10.1186/s12916-018-1106-7)
Supplement: Supplementary file 4 — Modelling Spending Rate (in 2014), clustered by CCG, sex/age variables. (DOCX 14 kb) [file 12916_2018_1106_MOESM4_ESM.docx]

File name: Additional file 4

File format: Docx

Title of the data: Modelling Spending Rate (in 2014), clustered by CCG, gender/age variables

| Modelling Spending Rate (in 2014), clustered by CCG, gender/age variables | | | |
| --- | --- | --- | --- |
| **Model predictors** |  | **Unadjusted point estimate (CI)** | **Adjusted* point estimate (CI)** |
| *Gender* |  |  |  |
|  | Change per % increase in female patients | 1.03 (1.02-1.04) | 1.00 (0.99-1.01) |
| *Age* |  |  |  |
|  | Change per % increase in patients <= 18 years | 0.99 (0.99-1.00) | 1.02 (1.02-1.03) |
|  | Change per % increase in patients >= 75 years | 1.06 (1.05-1.07) | 1.07 (1.06-1.07) |
| *Level of rurality* |  |  |  |
|  | Baseline: Rural | 1 | 1 |
|  | Towns and Cities | 0.85 (0.81-0.90) | 0.91 (0.84-0.98) |
|  | Urban Conurbation | 0.73 (0.67-0.80) | 0.87 (0.77-0.98) |
| *Level of deprivation* |  |  |  |
|  | Baseline: 1 - Least Deprived | 1 | 1 |
|  | 2 | 0.88 (0.83-0.92) | 0.88 (0.80-0.97) |
|  | 3 | 0.80 (0.76-0.85) | 0.91 (0.80-1.03) |
|  | 4 | 0.71 (0.67-0.75) | 0.83 (0.68-1.01) |
|  | 5 - Most Deprived | 0.67 (0.63-0.71) | 0.90 (0.67-1.29) |
| *Interaction between rurality and deprivation* | Towns and Cities - 2  Towns and Cities - 3  Towns and Cities - 4  Towns and Cities - 5  Urban Conurbation - 2  Urban Conurbation - 3  Urban Conurbation - 4  Urban Conurbation - 5 |  | 1.08 (0.97-1.22)  1.01 (0.88-1.16)  0.99 (0.80-1.21)  0.88 (0.61-1.27)  1.04 (0.97-1.19)  0.92 (0.79-1.08)  0.93 (0.75-1.16)  0.83 (0.57-1.21) |
| **Note:** CI = 95% confidence interval, * = Adjusted for all other predictors in the model | | | |
