# Supplementary material for: Breaking bread: examining the impact of policy changes in access to state-funded provisions of gluten-free foods in England
Source: BMC Med. 2018 Aug 2;16:119. doi: 10.1186/s12916-018-1106-7 (PMC6090920; doi:10.1186/s12916-018-1106-7)
Supplement: Supplementary file 5 — Mean and standard deviations for expenditure on GF products among CCGs with three policy types. (DOCX 13 kb) [file 12916_2018_1106_MOESM5_ESM.docx]

## Additional file 5

File name: Additional file 5

File format: Docx

Title of the data: Mean and standard deviations for expenditure on GF products among CCGs with three policy types

| **CCG Policy types (sample size)** | **3 months before policy change** | **2 months before policy change** | **1 month before policy change** | **Month of policy change** | **1 month after policy change** | **2 months after policy change** | **3 months after policy change** |
| --- | --- | --- | --- | --- | --- | --- | --- |
| **No ban** (n = 24) |  |  |  |  |  |  |  |
| Mean | 9062.763 | 9050.008 | 9255.055 | 9103.575 | 8829.318 | 8824.074 | 8838.009 |
| SD | 5083.342 | 4858.211 | 5356.859 | 4681.272 | 4952.351 | 4822.395 | 5067.913 |
| **Complete ban** (n = 24) |  |  |  |  |  |  |  |
| Mean | 10580.13 | 11047.3 | 11699.37 | 7718.281 | 3286.61 | 1997.549 | 1659.364 |
| SD | 6668.944 | 7262.868 | 8026.371 | 8519.755 | 4141.83 | 2803.1 | 2634.069 |
| **Complete ban, with age related exceptions** (n = 8) |  |  |  |  |  |  |  |
| Mean | 10971.63 | 11086.62 | 11444.08 | 7232.373 | 2865.018 | 2271.661 | 2152.043 |
| SD | 2979.162 | 2678.676 | 2675.779 | 3234.73 | 1441.856 | 987.4532 | 841.5215 |
| **Note:** SD = Standard deviation | | | | | | | |
